# Cinacalcet and primary hyperparathyroidism: systematic review and meta regression

**DOI:** 10.1530/EC-20-0221

**Published:** 2020-07-03

**Authors:** Cheng Han Ng, Yip Han Chin, Marcus Hon Qin Tan, Jun Xuan Ng, Samantha Peiling Yang, Jolene Jiayu Kiew, Chin Meng Khoo

**Affiliations:** 1 Yong Loo Lin School of Medicine, National University of Singapore, Singapore; 2 Department of Medicine, National University Hospital, Singapore

**Keywords:** cinacalcet, meta-analysis, primary hyperparathyroidism, hypercalcemia

## Abstract

**Purpose:**

Primary hyperparathyroidism (PHPT) is a common condition affecting people of all ages and is mainly treated with parathyroidectomy. Cinacalcet has been widely used in secondary or tertiary hyperparathyroidism, but the use of cinacalcet in PHPT is less clear.

**Methods:**

Searches were conducted in Medline and Embase for cinacalcet use in PHPT from induction to 10 April 2020. Articles and conferences abstracts describing the use of cinacalcet for PHPT in prospective or retrospective cohorts and randomized controlled trials restricted to English language only. We initially identified 1301 abstracts. Each article went extraction by two blinded authors on a structured proforma. Continuous outcomes were pooled with weight mean difference (WMD). Quality of included articles was assessed with Newcastle Ottwa Scale and Cochrane Risk of Bias 2.0.

**Results:**

Twenty-eight articles were included. Normalization rate of serum Ca levels was reported at 90% (CI: 0.82 to 0.96). Serum levels of Ca and PTH levels were significantly reduced (Ca, WMD: 1.647, CI: −1.922 to −1.371; PTH, WMD: −31.218, CI: −41.671 to −20.765) and phosphate levels significantly increased (WMD: 0.498, CI: 0.400 to 0.596) after cinacalcet therapy. The higher the baseline Ca levels, the greater Ca reduction with cinacalcet treatment. Age and gender did not modify the effect of cinacalcet on serum Ca levels.

**Conclusion:**

The results from the meta-analysis support the use of cinacalcet as an alternative or bridging therapy to treat hypercalcemia in people with PHPT.

## Introduction

Primary hyperparathyroidism (PHPT) is a common endocrine disorder that is caused by excessive or inappropriate parathyroid hormone (PTH) secretion with simultaneous derangement of both phosphate and calcium metabolism. PHPT is more prevalent in both elderly and female patients (1) and is mainly caused by parathyroid adenoma, but can also be due to parathyroid hyperplasia, parathyroid carcinoma, and rare genetic abnormalities such as multiple endocrine neoplasia (MEN) syndrome. Patients with PHPT might present with cardinal signs of hypercalcemia with significant involvement from both renal and skeletal system presenting with recurrent nephrolithiasis, fragility fractures, or both (2).

Parathyroidectomy is the current gold standard treatment for PHPT with resolution in hypercalcemia and hypophosphataemia. However, there is a subset of patients who might not be suitable candidates for surgery, or have refractory hypercalcemia despite parathyroidectomy, or prefer non-surgical intervention. Also, surgery might be delayed due to unforeseen circumstances, for example, the recent COVID-19 pandemic has resulted in postponement of non-essential surgeries such as parathyroidectomy. Thus, an effective non-surgical option as a bridging therapy to parathyroidectomy would be required to control hypercalcemia while awaiting surgery.

Cinacalcet is a positive allosteric modulator of the calcium sensing receptor (CaSR) that increases the sensitivity of the CaSR on the parathyroid glands, thereby reducing PTH secretion and serum Ca levels (3). Cinacalcet is widely used in patients with secondary or tertiary hyperparathyroidism. Its benefit in patients with PHPT is less known. Here, we conducted a meta-analysis alongside a case series to pool evidence in the use of cinacalcet in controlling hypercalcemia from PHPT.

## Materials and methods

### Search strategy

We adhered to the PRISMA guidelines of the synthesis of this review (4). Searches were conducted on 5 April 2020 on electronic database Medline and Embase. Keywords and thesaurus terms were used in the search for ‘Cinacalcet’ and ‘Primary hyperparathyroidism’ and abstracts were complied with duplicates removed in Endnote X9 (Supplementary Table 1, see section on supplementary materials given at the end of this article).

### Selection criteria and outcomes

The meta-analysis focuses on the use of cinacalcet in the treatment of hypercalcemia due to PHPT including primary adenoma and multiple endocrine neoplasia syndromes (MENS). We excluded the use of cinacalcet for parathyroid carcinoma and secondary or tertiary hyperparathyroidism in the context of chronic kidney disease. A variety of study designs were included including randomized controlled trials (RCTs), prospective and retrospective single arm cohort studies. Demographic data (sample size, age, gender, medical conditions) regarding interventional population were extracted. The main outcomes included serum Ca, PTH and phosphate before and after cinacalcet treatment, as well as the rate of normalization in Ca and PTH levels. When studies do not report the mean and s.d., transformation of the data was conducted through prevailing formulas (5, 6, 7). We also reported the reasons for prescribing cinacalcet, discontinuation rate and rationale, and adverse reactions related to cinacalcet treatment.

### Statistical analysis and quality assessment

Three type of analyses were conducted with the collected data. First, a meta-analysis of proportion was undertaken for binary data after a Freeman–Turkey double arcsine transformation to stabilize variance before analysis was pooled with DerSimonian and Laird random effects (8, 9). For continuous variables on the laboratory parameters of only post-cinacalcet use, the inverse variance method was used in pooling proportions. Next, pairwise comparisons were conducted with DerSimonian and Laird random effects regardless of heterogeneity measures (I^2^, Cochran Q test and Tau) for laboratory parameters between before and after cinacalcet use and cinacalcet compared to placebo (9). Continuous data were pooled with weight mean difference (WMD). Lastly, meta regression with random effects restricted maximum likelihood model was used to explore heterogeneity when sufficient data were available (*n* ≥ 10) (10). Knapp–Hartung variance estimator was used in the readjustment of variance (11). Publication bias was explored with Egger’s regression test (12). Statistical significance was considered when *P* < 0.05. Quality assessment of included articles was done by the Newcastle Ottawa scale for non-randomized studies, and randomized controlled trial (RCTs) was done with the Cochrane Risk of Bias 2.0 tool (13, 14). Visual representation of the risk of bias was done through the *robvis* tool (15).

## Results

### Meta-analysis

#### Literature review

**Figure 1:**
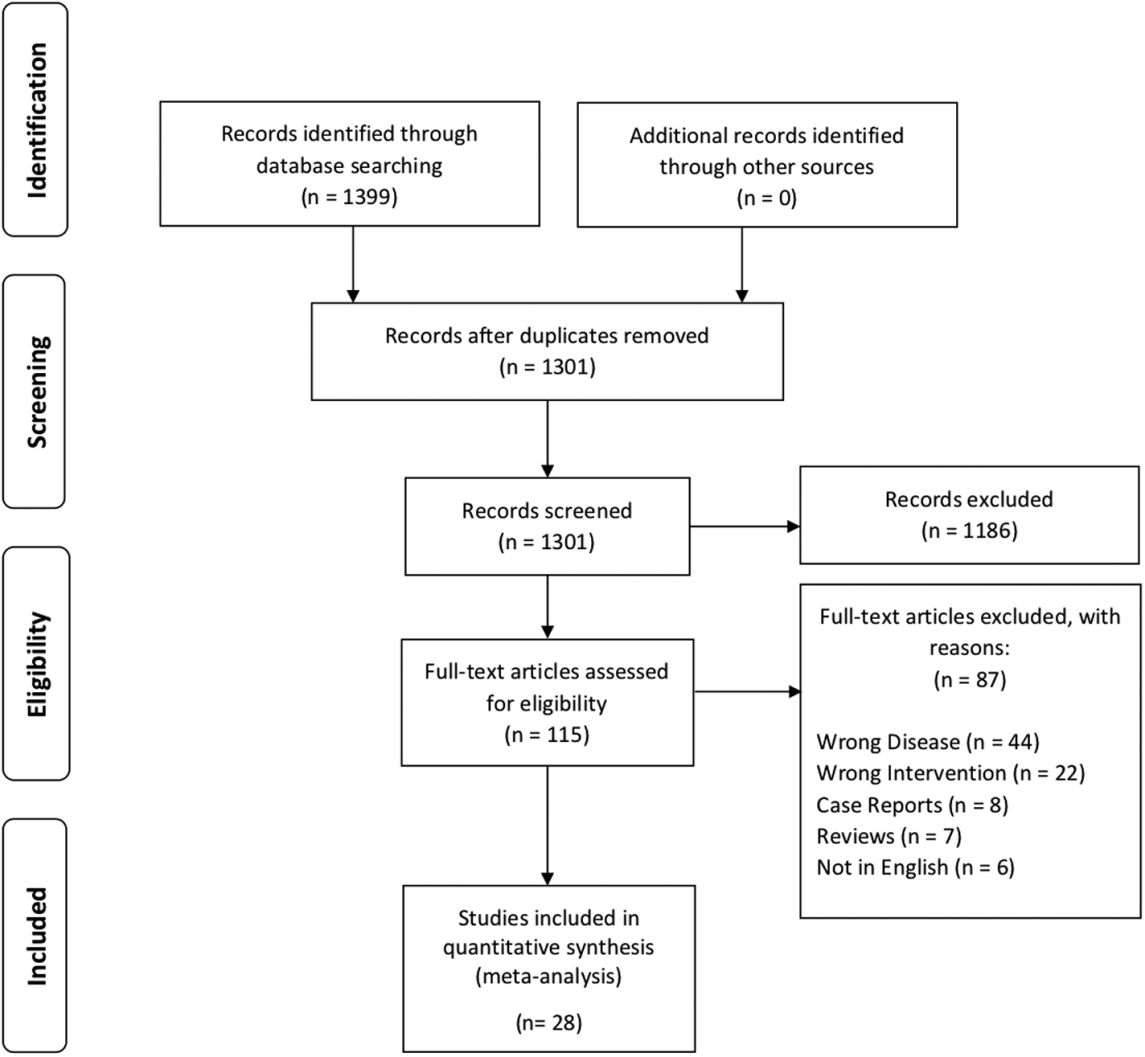
PRISMA flowchart.

**Figure 2:**
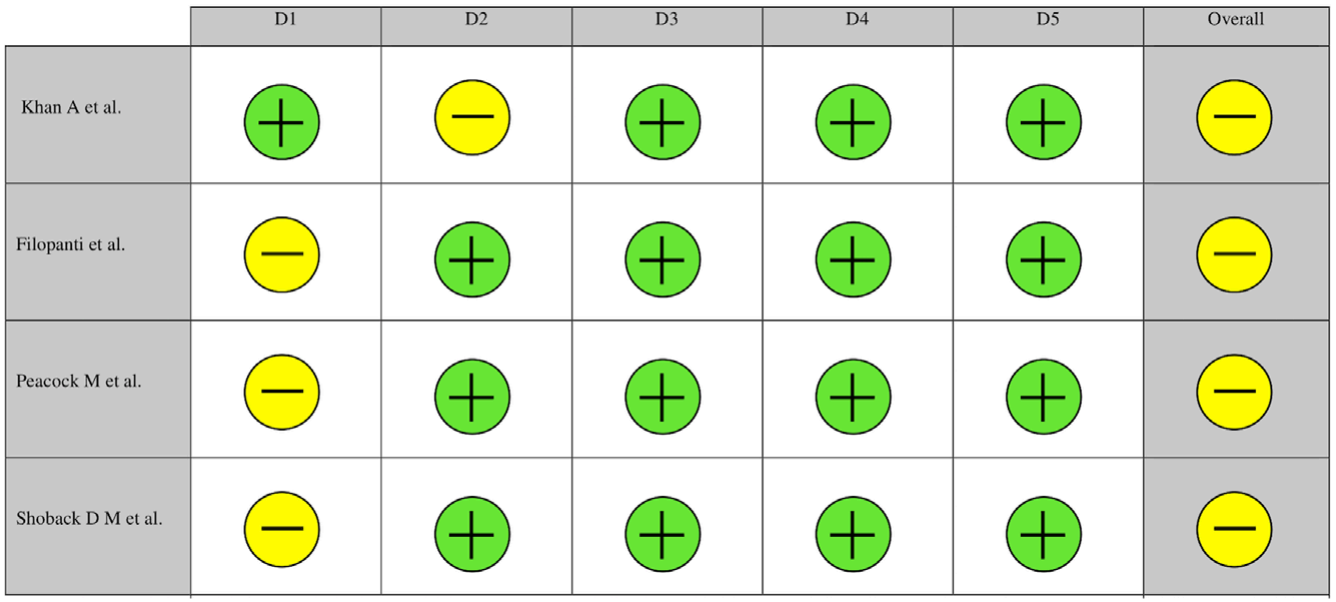
Cochrane risk of bias assessment of included articles.

A total of 1301 articles were identified after duplicates removal, and eventually 28 articles were included in the review (16, 17, 18, 19, 20, 21, 22, 23, 24, 25, 26, 27, 28, 29, 30, 31, 32, 33, 34, 35, 36, 37, 38, 39, 40, 41, 42, 43), of which 8 were conference proceedings (20, 24, 25, 30, 31, 32, 41, 42) (Fig. 1). In total, 823 patients underwent treatment with cinacalcet and 722 completed treatment for either PHPT (16, 17, 18, 20, 21, 22, 23, 24, 25, 26, 27, 28, 29, 30, 31, 33, 34, 35, 36, 37, 38, 39, 40, 41, 42, 43) or MENS (19, 24, 26, 28, 29, 32). A collective total of 101 patients did not complete treatment. Majority of included articles use cinacalcet as a monotherapy, while a minority (*n* = 9) had subsets of patients with adjunct medications (17, 21, 22, 24, 26, 29, 33, 39, 41). Four articles were randomized controlled trials comparing cinacalcet with placebo (22, 28, 37, 38), while majority were prospective and retrospective cohort studies. Majority of studies were conducted in America and Europe with exception to two studies (16, 17) (Japan and Israel). Table 1 summarizes the characteristics of included articles and the risk for bias for RCTs is presented in Fig. 2.

**Table 1:** Summary of included articles.

Author	Year	Study design	Sample size (studied/recruited)	Mean age	Condition	Dosing regimen	Other medications	NOS score
Duskin-Bitan *et al*.	2020	Retrospective	15/15	72.7	PHPT	Average dose of cinacalcet was 39 ± 14 mg per day.	NA	5
Manaka *et al*.	2019	Retrospective	61/61	67.8	PHPT	The mean cinacalcet maintenance dose was 43.4 mg with most patients maintained at 25–50 mg per day.	Twenty-six with bisphosphonates, denosumab or estrogen receptor modulators	6
Koman *et al*.	2019	Retrospective	101/110	62	PHPT	Patients were started with 30 mg of cinacalcet daily and were monitored closely by serum ionized Ca levels once weekly. Cinacalcet dose was increased to 60 mg if hypercalcemia persists after 2 weeks of treatment with cinacalcet 30 mg.	NA	5
Abusahmin *et al.*	2018	Prospective	11/11 18/18	63 85	PHPT PHPT	Cinacalcet was started at 30 mg once daily and gradually titrated upwards every 4–6 weeks, aiming for normal serum adjusted Ca levels.	Patients were also treated with Vitamin D.	4
Misiorowski *et al*.	2017	Prospective	21/23	56.9	PHPT	Cinacalcet was started twice daily with 30 mg and increased sequentially every week, depending on the serum Ca levels of the patient during the previous week and the adverse event evaluation. The dose was increased until the correct serum Ca level was below 11.3 mg/dL, the highest dose of 90 mg was reached four times daily or the patient experienced an adverse effect associated with increased dosage.	NA	5
Garcia *et al*.	2016	Retrospective	26/26	NA	PHPT	Cinacalcet was initially started at 30 mg daily and subsequently increased to 90 mg per day depending on the patient’s serum Ca levels and tolerance.	NA	4
Guisti *et al*.	2015	Prospective	28/33	40	MEN1 PHPT	Patients were started on cinacalcet 30 mg daily and increased to a maximum of 60–90 mg daily if patients had inadequate response to the previous dose for a period of 12 months.	NA	5
Khan *et al*.	2015	RCT	27/33	69.5	PHPT	Cinacalcet was started 30 mg twice daily and increased sequentially to 60 mg twice daily, 90 mg twice daily or 90 mg thrice daily. In order to maintain normal serum Ca levels, cinacalcet dosing may be altered every 4-week interval during the efficacy assessment phase.	Bisphosphonates in nine patients.	NA
Simone *et al*.	2015	Prospective	10/10	59	PHPT	The dosage of cinacalcet has been optimized to achieve a reduction of PTH and Ca levels within normal limits for each individual patient.	Patients were treated with hydrochlorothiazide 12.5 mg twice daily for 3 months before the study	5
Brardi *et al*.	2014	Retrospective	15/15	78.79	PHPT and MEN1 PHPT	NA	Vitamin D in nine patients; bisphosphonates in two patients.	5
Marotta *et al*.	2014	Retrospective	23/23 20/20	NA NA	Sporadic PHPT Sporadic PHPT	The study consisted of an initiation phase of 3 months, and a follow-up phase. Patients were started on cinacalcet four times daily during the initiation phase and no dose escalations were performed. Study visits and dose escalations were conducted every 3 weeks during the follow-up phase to achieve normocalcemia. Dose escalations were performed by sequential addition of 30 mg daily with the maximum dose of 90 mg four times allowed daily.	Treatment in combination with bisphosphonates was allowed. Treatment with 25OHVITD was also allowed but only in the follow-up phase.	5
Muñoz-Garach *et al*.	2014	Retrospective	27/27	NA	PHPT	NA	NA	4
Fernández *et al*.	2013	Prospective	20/34	67.15	PHPT and MEN PHPT	94% of patients were started on cinacalcet 30 mg every 12 h and 6% of patients were started on 30 mg once daily. The mean daily dose of cinacalcet was 60 mg with a range of 30180 mg.	76.4% of patients received treatment with 25-OH-D3 and 50% with bisphosphonates.	5
Norman *et al*.	2012	Prospective	51/70	60	PHPT	Cinacalcet was prescribed twice daily and titrated depending on serum Ca levels with the most frequent maintenance dose of 60 mg twice daily, followed by 90 mg daily in divided doses. The dose ranged from 60 to 120 mg per day.	NA	4
Filopanti *et al*.	2012	RCT	11/15 20/20	42.3 61	MEN1 PHPT Sporadic PHPT	Cinacalcet was started 30 mg daily and titrated after a week with addition of 30 mg until normal serum Ca levels were achieved. After titration, the dose was kept constant and maintained for 3 months. Sporadic PHPT patients were started on cinacalcet 30 mg daily and addition of 30 mg until normal serum Ca levels were achieved.	All patients were treated with 300,000 units of oral cholecalciferol every 4–6 months and stopped 1–2 months before the study began. 13 sporadic PHPT patients regularly took bisphosphonates for 9–48 months before the study.	NA
Cetani *et al*.	2012	Prospective	14/14	69.5	PHPT and MEN1 syndrome	Depending on patient’s serum Ca levels, cinacalcet was started 30 mg once daily if serum Ca levels were below 11.5 mg/dL or started 30 mg twice daily if serum Ca levels were above 11.5 mg/dL.	Bisphosphonates in eight patients.	4
Vai *et al*.	2011	Prospective	15/20	69.3	PHPT	Cinacalcet was administered 30 mg twice daily.	NA	4
Trombetti *et al*.	2011	Prospective	30/30	63.2	PHPT	The median start dose of cinacalcet was 30 ± 30 mg daily and subsequently adapted to 30–420 mg.	NA	5
Author	Year	Study design	Sample size (studied/recruited)	Mean age	Condition	Dosing regimen	Other medications	NOS score
Francesca *et al*.	2011	Prospective	7/7	46.1	MEN1 PHPT	NA	NA	4
Faggiano *et al*.	2011	Prospective	23/23	63.9	PHPT	Patients were started on cinacalcet 30 mg daily p.o. and increased to 30 mg at each assessment until normal serum Ca levels were achieved with the maximum dose allowed 90 mg daily.	Ten patients were treated in combination with alendronate.	5
Moyes *et al*.	2010	Retrospective	8/8	43.5	PHPT	Cinacalcet was administered 30 mg twice daily.	Patients with Vitamin D deficiency were started on supplements and achieved normal levels before the study began.	4
Faggiano *et al*.	2010	Prospective	14/14	64.1	PHPT	Cinacalcet was started at the dose of 30 mg daily after alendronate withdrawal.	Alendronate was used for the patients for 2 years. Alendronate was withdrawal for the patients followed by cinacalcet regimen.	4
Krajewska *et al*.	2009	Prospective	7/7	49	PHPT	Applied doses of cinacalcet ranges from 30 to 180 mg.	In combination with diuresis and/or bisphosphonates.	4
Peacock *et al*.	2009	Prospective	30/45	62.353	PHPT	All patients received cinacalcet 30 mg twice daily.	NA	5
Marcocci *et al*.	2009	Prospective	15/17	65.7	PHPT	Cinacalcet was started twice daily with 30 mg and increased sequentially every 2 weeks, depending on the serum Ca levels of the patient during the previous week and the adverse event evaluation. The dose increase continued until the correct serum Ca level was ≤10 mg/dL, the highest dose of 90 mg was reached four times daily or the patient experienced an adverse effect associated with increased dosage.	NA	5
Sajid-Crockett *et al*.	2008	Prospective	16/18	69.4375	PHPT	Cinacalcet was started 30 mg daily and the dose was adjusted every 2 weeks depending on the patient’s serum Ca levels.	NA	4
Peacock *et al*.	2004	RCT	27/40	62	PHPT	Patients were initially started on cinacalcet 30 mg twice daily and depending on the patient’s serum Ca levels, the dose was sequentially increased to 40 and 50 mg twice daily at study week 4 and 8.	NA	NA
Shoback *et al*.	2003	RCT	16/16	61	PHPT	Patients were randomized to receive cinacalcet twice daily with doses of 30 mg, 40 mg or 50 mg for 15 consecutive days.	NA	NA

‘NA’ annotates that the data is not available.

### Indications

The majority of indications for cinacalcet treatment are either for contraindication or refusal for surgery (19, 21, 22, 24, 26, 27, 28, 29, 31, 32, 33, 35, 36, 39, 43), reduction in serum Ca levels prior to surgery (18, 20, 26, 27, 31, 40, 41) or to treat refractory hypercalcemia despite parathyroidectomy (16, 19, 22, 27, 28, 29, 31, 32, 35, 36, 38, 41, 43).

### Effectiveness of cinacalcet treatment

#### Calcium (mg/dL)

**Figure 3:**
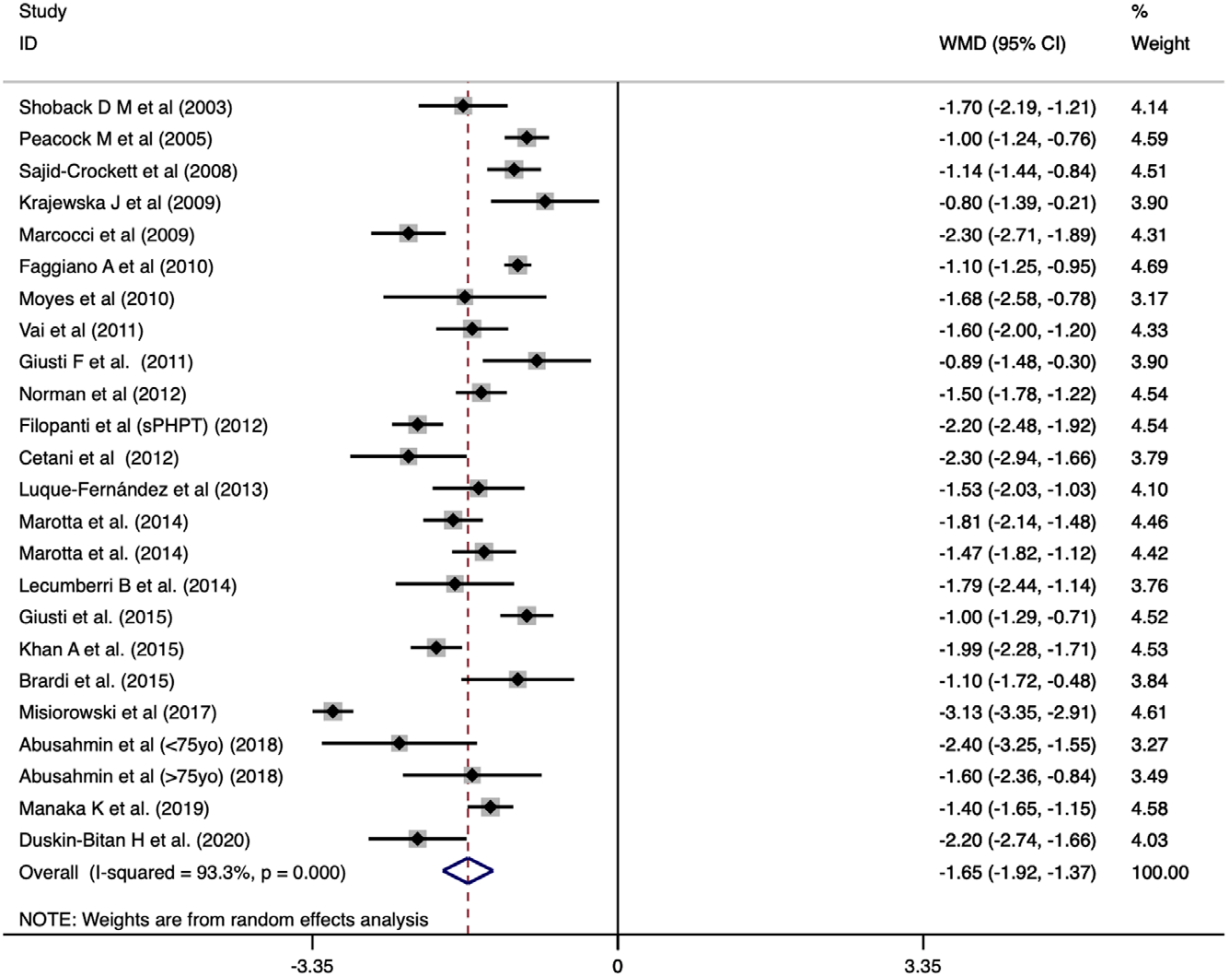
Forest plot of Ca levels before and after cinacalcet with Dersimonian and Laird random effects model.

With cinacalcet use, pooled proportions found that normalization rate of serum Ca was reported at 90% (CI: 0.82 to 0.96). Pooled analysis of serum Ca levels after cinacalcet treatment averaged at 9.733 (CI: 9.554 to 9.912). Comparing before and after cinacalcet treatment, cinacalcet significantly reduced the mean serum Ca level by 1.647 (CI: −1.922 to −1.371, *P* < 0.001, Fig. 3) from baseline. A subgroup analysis was done to examine the effect of cinacalcet between baseline values that was greater or smaller than 12 mg/dL. A larger mean reduction was observed (WMD: −2.501, CI: −2.994 to −2.009, *P* < 0.001, Fig. 2) when the baseline of Ca was ≥12 mg/dL compared to those <12 mg/dL (WMD: −1.437, CI: −1.629 to −1.245, *P* < 0.001, Fig. 3). The interaction was significant (*P* < 0.001) for the treatment effect between the baseline greater or smaller than 12 mg/dL. Publication bias was not statistically significant (*P* = 0.7804). Meta-regression was used to explore the relationship of effect with age and the proportion of females in patients with <12 mg/dL. Age (β = −0.0168, CI: −0.035 to 0.0017, *P* = 0.073) and proportion of females (β = −0.574, CI: −1.65 to 0.507, *P* = 0.274) were not statistically significant when meta regressed with Ca level difference. In the pooled analysis of 166 patients, cinacalcet treatment significantly reduced Ca levels (WMD: −1.65, CI: −2.01 to −1.26, *P* < 0.001) compared to placebo.

#### Parathyroid hormone (pg/mL)

**Figure 4:**
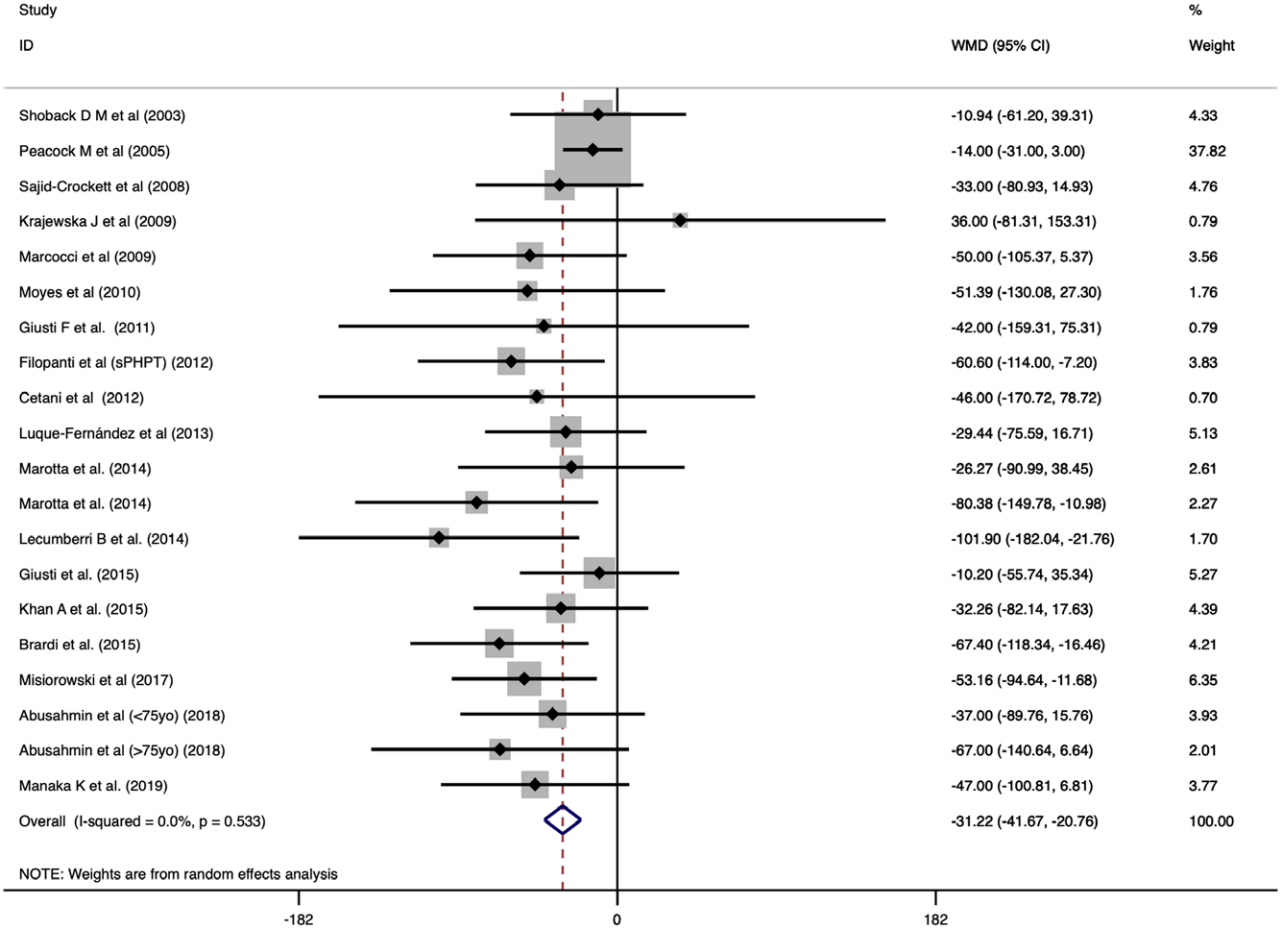
Forest plot of PTH levels before and after cinacalcet with Dersimonian and Laird random effects model.

Treatment with cinacalcet normalized PTH level in 10% (CI: 0.02 to 0.23) of the patients and the pooled mean post-treatment PTH levels was 95.276 pg/mL (CI: 83.131 to 107.420). The reduction of PTH was significantly different (WMD: −31.218, CI: −41.671 to −20.765, *P* < 0.001, Fig. 4). Publication bias by Egger’s regression was significant (*P* = 0.0211). Meta regression with age and gender was not significant (β = −0.9176, CI: −2.28 to 0.446, *P* = 0.187 and β = −2.264, CI: −71.67 to 67.14, *P* = 0.949). In the pooled analysis of 166 patients, cinacalcet significantly reduced PTH level (WMD: −26.796, CI: −39.647 to −13.945, *P* < 0.001) compared to placebo.

#### Phosphate (mg/dL)

**Figure 5:**
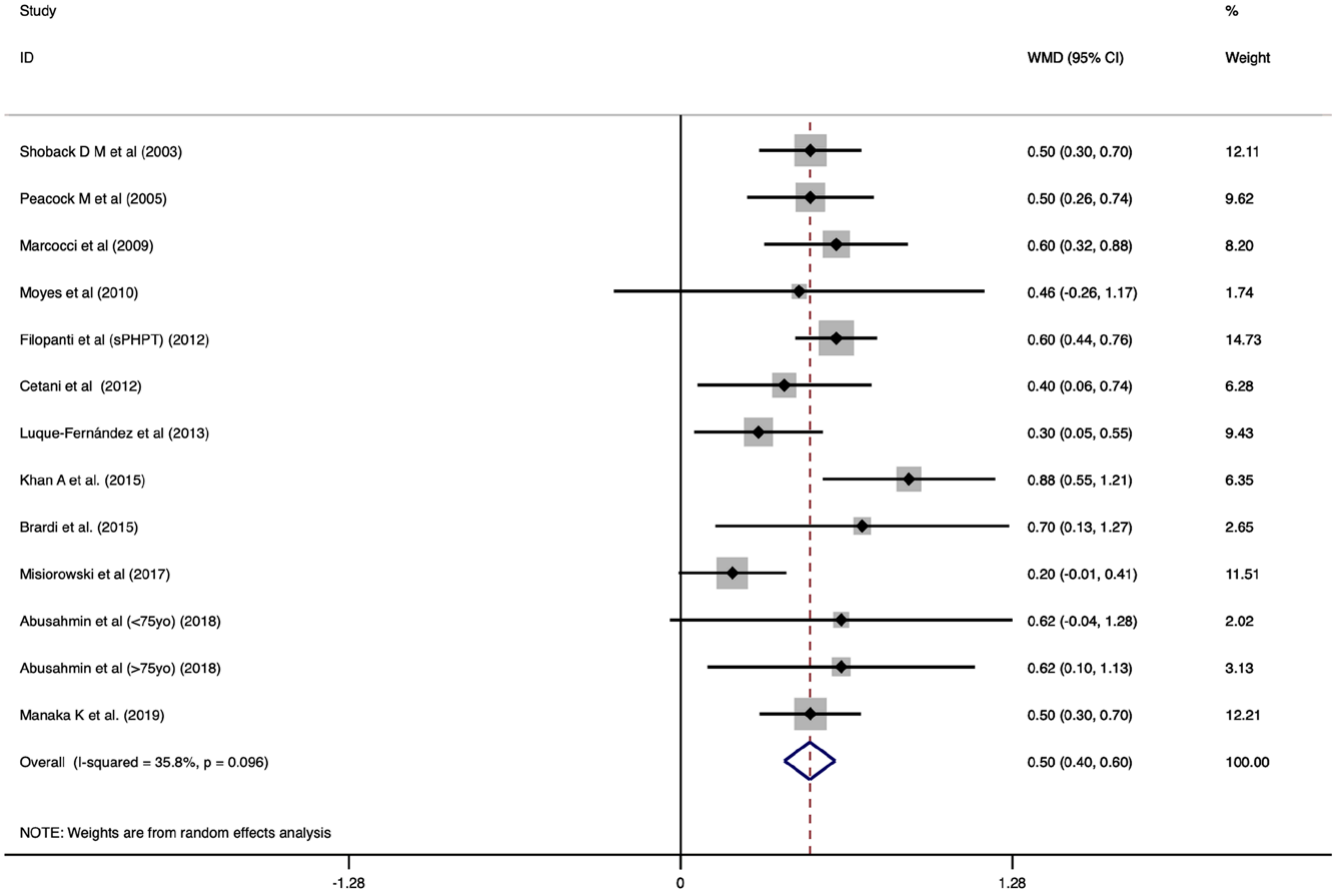
Forest plot of phosphate levels before and after cinacalcet with Dersimonian and Laird random effects model.

The mean phosphate level was 3.069 (CI: 2.882 to 3.256) after treatment with cinacalcet. The phosphate level significantly increased after cinacalcet treatment (WMD: 0.498, CI: 0.400 to 0.596, *P* < 0.001, Fig. 5). Publication bias was not statistically significant (*P* = 0.4589). Age and proportion of females did not modify the treatment effect of cinacalcet on phosphate levels (β = 0.009, CI: −0.0107 to 0.029, *P* = 0.327 and β = −0.294, CI: −0.690 to 1.278, *P* = 0.524, respectively). In the pooled analysis of 166 patients, cinacalcet significantly reduced phosphate levels (WMD: 0.634, CI: 0.445 to 0.824, *P* < 0.001) compared to placebo.

### Discontinuation and adverse reaction

In general, most adverse reactions were mild or moderate, mainly affecting the gastrointestinal system. The incidence rate of nausea or vomiting was 23% (CI: 0.14 to 0.33) and hypocalcemia was 3% (CI: 0.01 to 0.06). Most hypocalcemia cases reported were either asymptomatic or mild. However, one patient was referred to surgery after being hospitalized twice for symptomatic hypocalcemia (27). Paresthesia was also relatively uncommon with 19% (CI: 0.08 to 0.31) incidence. Other adverse reactions include muscle spasm/cramps (3.318.2%) (22, 31) and headache (23%) (37). Most patients were able to continue treatment despite the adverse reactions, and only a minority withdrew treatment (17, 18, 24, 25, 37).

## Discussion

To our best knowledge, this is the first meta-analysis of the use of cinacalcet in PHPT. The favourable outcomes on serum calcium, PTH and phosphate levels provide support of the use of cinacalcet in PHPT as a bridging therapy while awaiting surgery and as a potential non-surgical treatment option for PHPT. We recognise that parathyroidectomy is the mainstay therapy for most patients with PHPT. However, parathyroidectomy could result in persistent postoperative hypocalcemia and, for some patients, hypercalcemia persists. In elderly patients with PHPT, the higher operative risk from comorbidities might deem them not suitable for surgery or delay the timing to surgery (44, 45). Also, the success of parathyroidectomy depends on the pre-operative localization of the culprit parathyroid gland which can be challenging. Medical therapy for PHPT, such as calcitonin or anti-resorptive therapy (bisphosphonates or denosumab), has a modest effect on hypercalcemia level and is often short-lived (46).

In our meta-analysis, the use of cinacalcet significantly reduced the serum levels of Ca and normalization of Ca level was achieved in nearly 90% of patients. The reduction in Ca levels was accompanied by an increase in phosphate levels indicating that cinacalcet treatment can restore normal Ca homeostasis. Interestingly, Silverberg *et al*. observed that the largest reduction of serum Ca was in patients with higher baseline Ca levels (47). Our study agrees with Silverberg et al., where a larger treatment effect was observed for a higher baseline serum Ca (12.0 mg/dL or higher). This finding is relevant, as people with serum Ca 12 mg/dL or higher usually present with neurological symptoms of hypercalcemia such as confusion and altered mental status, which necessitates medical treatment. For these select patients, cinacalcet is a reasonable option in the treatment regimen to control hypercalcemia. Whether cinacalcet would have a place in the immediate medical treatment of severe PTH-dependent hypercalcemia would warrant further study. It is also comforting that age and gender do not modify the treatment efficacy of cinacalcet.

While cinacalcet reduces circulating PTH levels, we found that there was a lack of change in the size of the parathyroid gland as reported in two studies. Faggiano *et al*. utilized ultrasonographic examination every 6 months for the 24-month study and reported no change in the size of the parathyroid gland (33). Peacock *et al*. extrapolated that, with the relatively stable dose throughout the 5 years of study, the cell mass or secretory function was likely to be constant (34). The reduction in the parathyroid gland size was observed in the rat models of secondary hyperparathyroidism and in patients with secondary hyperparathyroidism where cinacalcet treatment prevents hyperplasia of the parathyroid gland (48, 49). The effect of cinacalcet on size control of parathyroid glands in PHPT has not been studied. However, usually parathyroid adenoma or hyperplasia does not cause local symptoms, hence the benefits of cinacalcet in parathyroid gland size control is less critical.

Our meta-analysis shows that normalisation in PTH levels after cinacalcet therapy only occurred in 10% of patients. In contrary, surgical treatment (parathyroidectomy) has reported 63% normalization rates (50). There are several explanations to this difference. A likely mechanism explaining the lack of normalization in cinacalcet can be attributed to the physiological properties of PTH. The PTH is subjected to significant variation, affected by circadian, seasonal and pulsatile ultradian secretion (51). The secretion pattern can be altered in PHPT, with an increase in basal secretion and total PTH secreted every hour (52). Measurements of PTH levels which are typically done at fasting state can result in the underestimation of actual normalization rates. The 24-h PTH levels would provide a better estimate on the PTH levels, though impractical. The lack of normalization of PTH with cinacalcet could also be due to increased secretion of PTH peptide fragments (active or inactive) from increased intracellular degradation of PTH. Also, cinacalcet treatment is usually titrated to improve hypercalcemia to a level that is less harmful to patients, but not toward the normalization of PTH level. Nonetheless, the lack of PTH suppression argues against the long-term use for PHPT due to unopposed PTH effects on skeletal complications. The effects of cinacalcet on fracture risk, urinary Ca excretion, and kidney stones are less clear, but less critical if cinacalcet is used as bridging therapy. However, these information including longitudinal quality-of-life measures would be important if cinacalcet is used as a long-term alternative to surgery to control hypercalcemia in mild to moderate PHPT. The use of cinacalcet for patients with PHPT in combination with anti-resorptive to control hypercalcemia and to improve skeletal health from elevated PTH level would warrant further investigations.

Regarding cinacalcet adverse reactions, the reported cases are relatively small. The majority were classified as mild to moderate severity and were relatively uncommon to result in withdrawal from treatment. Nausea or vomiting accounted for the largest adverse event reported, occurring in 23% (CI: 0.14 to 0.33) of the population studied which can be treated with anti-emetics. However, care should be taken to avoid drugs that can prolong the QT interval in the setting of possible hypocalcemia (53). Other adverse drug reactions included hypocalcemia (3% CI: 0.01 to 0.06), paresthesia (19%, CI: 0.08 to 0.31) and muscle spasm. Although rare, severe hypocalcemia after cinacalcet has been associated with higher baseline serum PTH levels (54). It may be a result of over suppression of PTH or abrupt lowering of PTH secondary to enhanced activation of CaSR (53). As a whole, hypocalcemia experienced after cinacalcet is generally mild and asymptomatic, and dose adjustment of cinacalcet is generally sufficient to prevent severe hypocalcemia. Importantly, the use of cinacalcet does not guarantee hypercalcemia treatment success, although there are cases of spontaneous remission of PHPT reported by Manaka *et al*. and Ferrari *et al*. (17, 55).

### Limitations

There are several limitations to this review. First, we only synthesized English language literature. Next, we included conference abstracts in the meta-analysis. This decision was made based on small sample sizes on the use of cinacalcet in the treatment of PHPT. While some have argued against the inclusion of grey literature in meta-analysis, the Cochrane Group recommends the inclusion of grey literature to reduce potential publication bias (56, 57). Additionally, the long-term use of cinacalcet as medical treatment for PHPT would need to consider the cost of cinacalcet and the long-term safety data.

## Conclusions

In conclusion, this meta-analysis provides support for the use of cinacalcet as medical option to control hypercalcemia in patients with PHPT. The use of cinacalcet as a long-term treatment for patients who are not eligible for parathyroidectomy, patients with persistent disease after parathyroidectomy, or patients who decline parathyroidectomy is an interesting proposition and would require further investigations and cost consideration.

## Supplementary Material

Supplementary Table 1: Search Strategy for Medline

## Declaration of interest

The authors declare that there is no conflict of interest that could be perceived as prejudicing the impartiality of the research reported.

## Funding

This research did not receive any specific grant from any funding agency in the public, commercial or not-for-profit sector.

## Author contribution statement

C M K and S P Y performed study conception and design. C H N, Y H C, M H Q T and J X N involved in the acquisition of data. C M K, S P Y, J J K and C H N contributed to the analysis and interpretation of data. C H N, Y H C, M H Q T, J X N, S P Y, J J K and C M K involved in drafting the manuscript. S P Y, J J K and C M K critically revised the manuscript.
