# Supplementary material for: Cinacalcet and primary hyperparathyroidism: systematic review and meta regression
Source: Endocr Connect. 2020 Jul 3;9(7):724–35. doi: 10.1530/EC-20-0221 (PMC7424342; doi:10.1530/EC-20-0221)
Supplement: Supplementary Table 1: Search Strategy for Medline [file supplementary_table_1.pdf]

Supplementary Table 1: Search Strategy for Medline

Hyperparathyroid	*hyperparathyroidism, primary/ or PHPT.tw. or ((primary or tertiary) adj3 (hyperparathyroidism)).tw. or (parathyroid adj3 (adenoma or hyperplasia or carcinoma)).tw. or MEN.tw. or multiple endocrine neoplasia*.tw.
Cinacalcet	exp Cinacalcet/ or (Cinacalcet or R 568 or AMG074 or AMG073 or KRN1493 or calcimimetic).tw.
NOT	(Case series or case report).tw. or (animals not humans).sh. or (systematic review or meta).ti.
